# NSFC-funded Chinese materia medica research (2015–2024): achievements output, research characteristics, and evolution

**DOI:** 10.3389/fphar.2026.1908693

**Published:** 2026-08-04

**Authors:** Hu Jin, Wang Can, Song Wei, Bai Hua, Wu Qunli

**Affiliations:** 1 Office of Scientific Research, Peking Union Medical College Hospital, Chinese Academy of Medical Sciences and Peking Union Medical College, Beijing, China; 2 Chinese Academy of Medical Sciences and Peking Union Medical College, Beijing, China; 3 Department of Traditional Chinese Medicine, Peking Union Medical College Hospital, Chinese Academy of Medical Sciences and Peking Union Medical College, Beijing, China

**Keywords:** Chinese materia medica, discipline development, national natural science foundation of China (NSFC), research characteristics, research translation, scientific research output

## Abstract

**Background:**

To systematically analyze the project completion status and research characteristics in the field of Chinese Materia Medica (CMM, discipline code H32) funded by the National Natural Science Foundation of China (NSFC), thereby providing a basis for optimizing the allocation of scientific research resources and adjusting funding strategies.

**Methods:**

Based on the NSFC big database system, a retrospective analysis was conducted to descriptively analyze the research output, potential translational capacity, and research characteristics of completed projects in the CMM field funded by NSFC from 2015 to 2024.

**Results:**

From 2015 to 2024, a total of 4,574 projects in the CMM field were completed, with a compound annual growth rate (CAGR) of 3.0%. The cumulative total funding was 1.86 billion RMB, with an average of 410 KRMB per project. The average research output was 6.6 items per project, and the potential patent conversion ratio was 61%. For the first time, the 19 sub-directions within the CMM field were categorized into four echelons: double-high advantage, high-output low-conversion, low-output high-conversion, and Low-Output Low-Conversion areas. Key advantage areas included CMM pharmacodynamic substances, CMM resources, and ethnic pharmacology. Disease research primarily focused on cardiovascular and cerebrovascular diseases, tumors, metabolic diseases, and neurological disorders. Technical methodologies rapidly evolved from traditional chemical analysis to multi-omics and systems biology, while research mechanisms expanded towards multi-dimensional regulatory networks.

**Conclusion:**

NSFC funding for the CMM field has been consistently stable. The discipline demonstrates an overall trajectory characterized by deepening basic research, rapid iteration of technical methods, increasing diversification of research directions, and steadily improving translational potential. These findings provide data support and decision-making references for optimizing NSFC funding strategies in the CMM field.

## Graphical Abstract

**Figure:**
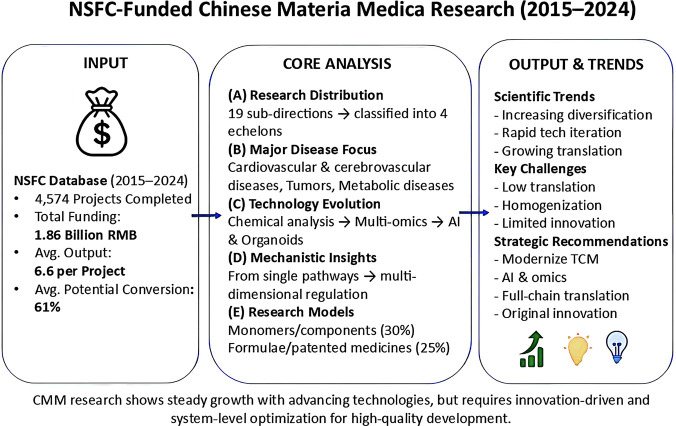

## 1 Introduction

The National Natural Science Foundation of China (NSFC) provides financial support and an innovation platform for researchers, serving as the core primary channel for supporting basic research in China. Its project portfolio and development trends directly reflect the alignment between disciplinary advancement and national strategic needs (Ma et al., 2011). Chinese Materia Medica (CMM) is a core component of traditional Chinese medicine (TCM) and represents the crystallization of the Chinese nation’s wisdom in disease prevention and treatment over millennia. It is also a uniquely advantageous area within China’s medical and health system (Wen et al., 2023; Zhao et al., 2025). In 2023, the General Office of the State Council issued the “Implementation Plan for the Major Project to Revitalize TCM,” explicitly emphasizing the need to strengthen basic research and key technological breakthroughs in TCM.

In recent years, many scholars have conducted a series of relevant studies on NSFC-funded projects in the CMM field, covering various sub-directions such as CMM resources, CMM identification, CMM pharmacodynamic substances, and CMM pharmacology (Gao et al., 2016; Shang et al., 2011; Wang et al., 2016; Chen et al., 2014; Han et al., 2022; Peng et al., 2012; Cai et al., 2025; Tan et al., 2021). However, overall, most previous studies have focused on NSFC project application and funding status, development characteristics of single sub-directions, or bibliometric analyses of research hotspots (Wu et al., 2025; Shen et al., 2022; Chen Y. H. et al. 2025; Chen et al., 2022; Dai and Liao, 2021). Systematic analyses focusing on the research output characteristics, translational potential, and in-depth research features of completed projects have not yet been reported. The authors believe that funding data only reflect resource input, whereas the research output and translational potential of completed projects are the core indicators for measuring the quality of disciplinary development and the efficiency of funding. Therefore, this study, based on data from completed NSFC projects in the CMM field from 2015 to 2024 and their research outputs, conducts in-depth analyses from the perspectives of overall completion status, research output and potential translation across sub-directions with four-echelon classification, research characteristics (including disease types, technologies and methods, research mechanisms and models, and characteristics and trends across different periods), as well as recent frontiers and hotspots. The goal is to more clearly grasp the current research development dynamics and output status of this field, providing data support for optimizing the allocation of scientific research resources and adjusting funding strategies.

## 2 Materials and methods

### 2.1 Data source

Data were obtained from the NSFC Big Data Knowledge Management Service Database (https://kd.nsfc.cn/), with completion information updated through 2024 (as of 26 May 2026) (National Natural Science Foundation of China, 2025). Retrospective retrieval was conducted for information on completed projects from 2015 to 2024 in the Department of Health Sciences (code H), the discipline of CMM (code H32), and its 19 sub-directions (H3201-H3,219), including the number of completed projects, research topics, keywords, abstracts, funding types, funding amounts, supporting institutions, and categories and quantities of research outputs. The number of completed projects includes General Projects, Young Scientist Fund Projects, Fund for Less Developed Regions Projects, Joint Fund Projects, and Key Projects. Categories of research output include journal articles, conference papers, patents, awards, and monographs, which are all derived from NSFC Big Data Knowledge Management Service Database. The total research output quantity is the cumulative number of these output categories. All counted patents are domestic and overseas patents archived by all completed NSFC projects, including pending patent applications and granted patents.

### 2.2 Analytical methods

GraphPad Prism 10.0 software was used for data analysis. Compound Annual Growth Rate (CAGR) = ((final value/initial value)^(1/n) − 1)^ × 100%, where n is the number of years of growth. Research output ratio = total number of research outputs/number of completed projects, reflecting the average output capacity of projects. Potential conversion ratio = number of patents/number of completed projects (Zhang and Guo, 2013), serving only as a preliminary statistical proxy for translational potential. This indicator has inherent limitations: sheer patent quantity cannot represent real industrial or clinical transformation benefits. Using the average research output ratio of 6.6 and the average potential conversion ratio of 61% for the CMM field as reference benchmarks, the 19 sub-directions were categorized into four echelons.

The research characteristic analysis adhered to the following principles: (1) Double-independent annotation to ensure objectivity and consistency (Kappa test was used to evaluate annotation consistency; Kappa value = 0.87, indicating high consistency); (2) For analyses of diseases, technologies/methods, research mechanisms, and research models, projects without clear indications in their topics and keywords were excluded. A standardized dictionary containing 655 synonyms was established to uniformly categorize disease names, technologies/methods, research mechanisms, *etc.*

All botanical scientific names mentioned in this manuscript were verified against the latest taxonomic data of World Flora Online (http://www.worldfloraonline.org, accessed on 4 June 2026) and MPNS (http://mpns.kew.org).

## 3 Results

### 3.1 Overall situation

From 2015 to 2024, a cumulative total of 4,574 projects in the CMM field (H32) were completed, accounting for 5.1% of the total completed projects in the Department of Health Sciences. The average annual number of completed projects was 457, with a compound annual growth rate (CAGR) of 3.0%, showing a steady and sustained growth trend, albeit slightly lower than the average growth rate of the Department of Health Sciences (4.4%) (Table 1).

**TABLE 1:** Comparison of completed projects between CMM field and department of health sciences of NSFC from 2015 to 2024.

Code	Direction	2024	2015–2024			Cumulative Funding (10 KRMB)
		Number	Cumulative number	Average number (n/Year)	CAGR (%)	
H	Department of health sciences	10,795	90,151	9,015	4.4	-
H32	Chinese materia medica	517	4,574	457	3.0	186,128
	General projects	178	1,762	176	0.3	105,185
	Young scientist fund projects	223	1,854	185	4.0	40,906
	Fund for less developed regions projects	110	947	95	6.1	37,586
	Joint fund projects	5	7	1	-	1,361
	Key projects	1	4	0	-	1,090
	% (CMM/Dept. Of health sciences)	4.8%	5.1%	5.1%	68%	-

Due to the small number of projects, CAGR, for Joint Fund Projects and Key Projects was not calculated, indicated as “-”.

In terms of project funding types: General Projects accounted for 1,762 completed projects (38.5%, CAGR 0.3%) with cumulative funding of 1,051.85 million RMB (57%), representing a stable core supporting force for basic research in CMM; Young Scientist Fund Projects accounted for 1,854 completed projects (40.5%, CAGR 4.0%) with cumulative funding of 409.06 million RMB (22%), representing the largest number and fastest-growing funding type; Fund for Less Developed Regions Projects accounted for 947 completed projects (20.7%, CAGR 6.1%), with a growth rate significantly higher than that of General Projects, reflecting the NSFC’s continued support for basic research in CMM in less developed regions, effectively promoting balanced disciplinary development nationwide; Joint Fund Projects and Key Projects accounted for very low proportions, only 0.2% and 0.1%, respectively.

### 3.2 Comparison across different sub-directions

#### 3.2.1 Number of completed projects and funding amounts

From 2015 to 2024, the number of completed projects and funding amounts in the CMM field exhibited a significantly uneven distribution (Table 2). Regarding the number of completed projects, CMM Pharmacodynamic Substances (H3203) had 838 completed projects (18%), ranking first, reflecting that the discovery, isolation, identification, and activity evaluation of CMM pharmacodynamic substances remain the core direction of basic research in CMM. This was followed by CMM Resources (H3201) with 558 completed projects (12%), focusing on the authenticity, germplasm resources, resource evaluation, and sustainable utilization of CMM. Ethnic Pharmacology (H3218) ranked third with 489 completed projects (11%), reflecting the sustained activity in the development of characteristic ethnic medicine resources and validation of traditional efficacy, making it a uniquely important component of the CMM field.

**TABLE 2:** Completed project status of sub-directions in CMM field of NSFC from 2015 to 2024.

Code	Direction	Cumulative number	Average number (n/Year)	Proportion of projects (%)	Total funding (10 KRMB)	Average funding (10 KRMB)
H3201	CMM resources	558	56	12	22,995	41
H3202	CMM identification	97	10	2	4,376	45
H3203	CMM pharmacodynamic substances	838	84	18	35,623	43
H3204	CMM quality evaluation	208	21	5	8,483	41
H3205	CMM processing	187	19	4	7,906	42
H3206	CMM preparations	256	26	6	10,583	41
H3207	CMM property theory	134	13	3	5,876	44
H3208	CMM neuropharmacology	188	19	4	7,450	40
H3209	CMM cardiovascular and cerebrovascular pharmacology	264	26	6	10,532	40
H3210	CMM antitumor pharmacology	382	38	8	14,385	38
H3211	CMM endocrine and metabolic pharmacology	183	18	4	7,498	41
H3212	CMM anti-inflammatory and immunopharmacology	200	20	4	7,784	39
H3213	CMM antiviral and anti-infective pharmacology	63	6	1	2,374	38
H3214	CMM digestive and respiratory pharmacology	135	14	3	5,654	42
H3215	CMM urinary and reproductive pharmacology	47	5	1	1,703	36
H3216	CMM metabolism and pharmacokinetics	133	13	3	5,224	39
H3217	CMM toxicology	97	10	2	4,031	42
H3218	Ethnic pharmacology	489	49	11	19,032	39
H3219	New technologies and methods in CMM research	115	12	3	4,619	40
H32	CMM	4,574	457	100	186,128	41

Regarding funding amounts, the total cumulative funding for the 19 sub-directions in the CMM field from 2015 to 2024 was 1,861.28 million RMB, with an average funding of 410,000 RMB per project, indicating stable overall funding intensity. CMM Pharmacodynamic Substances (H3203), CMM Resources (H3201), and Ethnic Pharmacology (H3218) ranked top three. Their cumulative funding accounted for 41.7% of the total, highly consistent with the distribution pattern of completed project numbers. Regarding the average funding amount per direction, the average funding for each sub-direction ranged from 360 to 450 KRMB, with small overall differences.

#### 3.2.2 Research output and potential translation

From 2015 to 2024, the CMM field cumulatively produced 30,216 research outputs, with an average research output ratio of 6.6 per project. There is a total of 2,811 patents, with an average potential conversion ratio of 61% (Table 3).

**TABLE 3:** Research output status of completed projects in sub-directions of CMM field of NSFC from 2015 to 2024.

Echelon classification	Code	Cumulative outputs (n)	Output proportion (%)	Research output ratio	Cumulative patents (n)	Potential conversion ratio
High output-High conversion	H3219	770	3	6.7	105	91%
	H3218	3,327	11	6.8	404	83%
	H3201	3,733	12	6.7	460	82%
	H3203	6,030	20	7.2	650	78%
	H3206	2,009	7	7.8	180	70%
High output-low conversion	H3207	1,035	3	7.7	37	28%
	H3217	712	2	7.3	32	33%
	H3202	673	2	6.9	36	37%
	H3216	924	3	6.9	58	44%
	H3205	1,246	4	6.7	94	50%
Low output-High conversion	H3204	1,333	4	6.4	150	72%
	H3212	1,191	4	6	141	71%
Low output-low conversion	H3214	895	3	6.6	70	52%
	H3211	1,169	4	6.4	90	49%
	H3209	1,640	5	6.2	82	31%
	H3208	1,111	4	5.9	66	35%
	H3210	1,936	6	5.1	125	33%
	H3213	290	1	4.6	22	35%
	H3215	192	1	4.1	9	19%
Total	H32	30,216	100	6.6	2,811	61%

**FIGURE 1:**
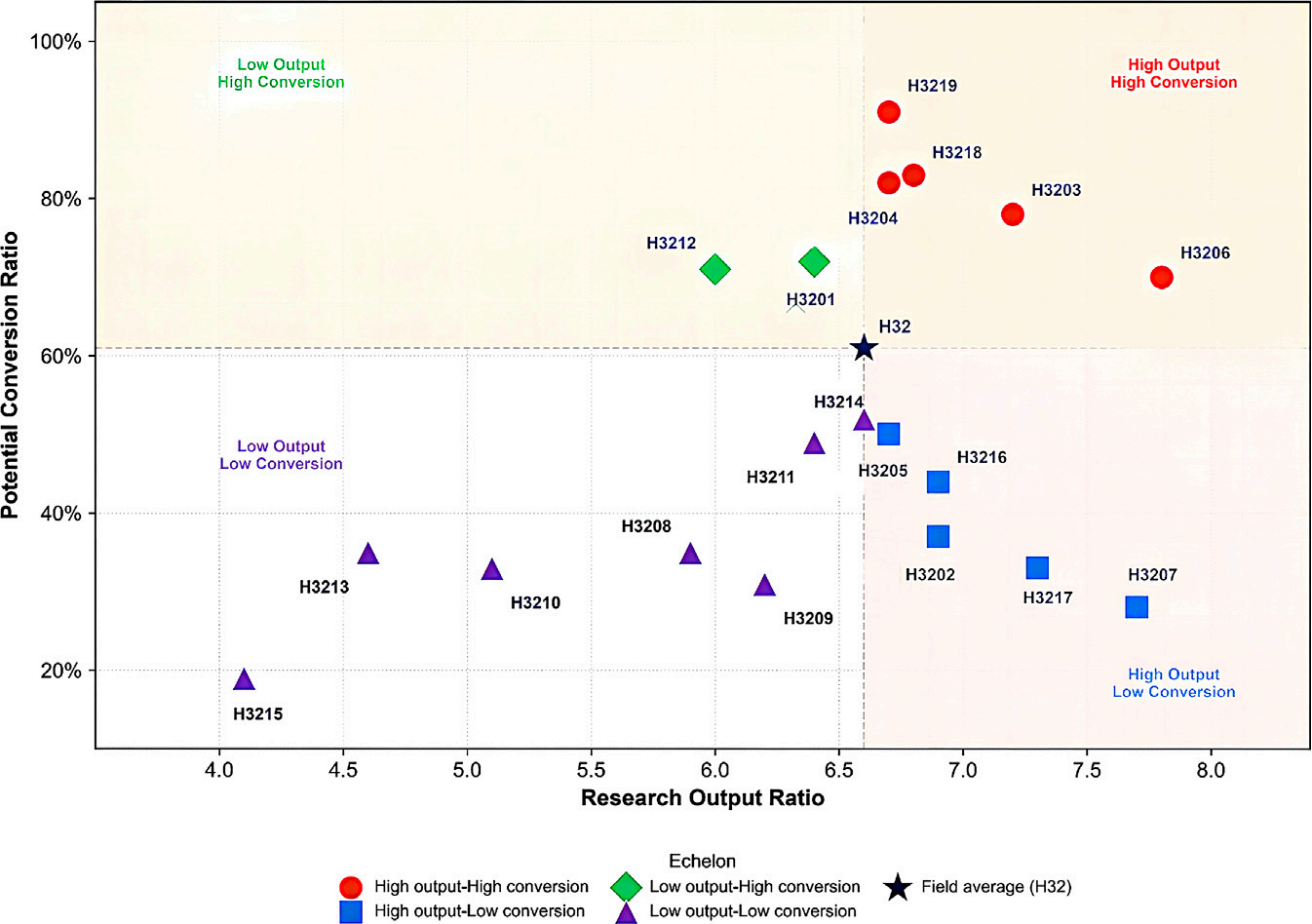
Four-echelon distribution of sub-directions in CMM field of NSFC from 2015 to 2024.

Based on the comprehensive research output and potential conversion ratio of each sub-direction, they were divided into four echelons (Figure 1): (1) High-Output High-Conversion Echelon: Leading in both basic research and translational potential, core support for disciplinary development. This echelon includes 5 sub-directions: New Technologies and Methods in CMM Research (H3219), Ethnic Pharmacology (H3218), CMM Resources (H3201), CMM Pharmacodynamic Substances (H3203), and CMM Preparations (H3206). They collectively contributed 43% of research outputs and over 60% of patents. The average research output ratio was 7.0, 0.4 higher than the field average; the average potential conversion ratio was 81%, 20% higher than the field average. (2) High-Output Low-Conversion Echelon: Outstanding academic output capacity but severely insufficient translational potential. This echelon includes 5 sub-directions: CMM Property Theory (H3207), CMM Toxicology (H3217), CMM Identification (H3202), CMM Metabolism and Pharmacokinetics (H3216), and CMM Processing (H3205). (3) Low-Output High-Conversion Echelon: Relatively weak basic research output capacity but high translational efficiency, representing potential areas for translation and crucial future growth points. This echelon includes 2 sub-directions: CMM Quality Evaluation (H3204) and CMM Anti-inflammatory and Immunopharmacology (H3212). (4) Low-Output Low-Conversion Echelon: Insufficient in both basic research output and translational potential, representing the core shortcomings for high-quality development in the CMM field. This echelon includes 7 sub-directions, such as CMM Antitumor Pharmacology (H3210), CMM Cardiovascular and Cerebrovascular Pharmacology (H3209), and CMM Neuropharmacology (H3208). The average research output ratio was 5.5, 1.1 lower than the field average; the average potential conversion ratio was 34%, 27% lower than the field average. These sub-directions are all related to pharmacological research across different systems. Although the total number of completed projects in these directions accounted for 36% of the total in the CMM field, high research homogenization, significant innovation challenges, and intense competition for publication in high-level journals resulted in low research output ratios and patent potential conversion ratios. This characteristic suggests that simply increasing the number of projects does not necessarily lead to high-quality outputs, and attention should be paid to the erosion of innovative vitality by “low-level” repetitive research.

### 3.3 Research characteristic analysis

Based on information from the research topics, keywords, and abstracts of completed projects, analyses were conducted categorizing disease types, technologies and methods, research mechanisms, research models, and research characteristics and trends across different periods. Considering the 1 to 3 years time lag in the completion data, this study also incorporates an extended analysis based on research frontiers and progress in CMM from 2024 to 2026, but these are not included in the quantitative statistics.

#### 3.3.1 Disease types

From 2015 to 2024, the disease types involved in completed projects in the CMM field showed significant concentration, with the top four core disease areas accounting for 75% of the total, representing the core research directions (Table 4). Cardiovascular and cerebrovascular diseases were the most concentrated research area, accounting for 25%, covering myocardial ischemia/reperfusion injury, cerebral ischemia/stroke, and atherosclerosis. Malignant tumors were the second largest research direction, accounting for 20%, covering liver cancer, lung cancer, breast cancer, and other high-incidence malignancies in China, focusing on the antitumor effects of CMM, toxicity reduction with efficacy enhancement, and reversal of drug resistance. Metabolic diseases and neuropsychiatric disorders had comparable research scales, each accounting for 15%. The former mainly covered type 2 diabetes mellitus and non-alcoholic fatty liver disease, while the latter focused on Alzheimer’s disease, Parkinson’s disease, and depression, all representing rapidly growing research directions in recent years. Additionally, autoimmune/inflammatory diseases, infectious diseases, and other diseases accounted for 13%, 7%, and 5%, respectively.

**TABLE 4:** Analysis of disease types involved in completed projects in CMM field of NSFC from 2015 to 2024.

Disease type	Number of projects (n)	Proportion (%)	Representative diseases
Cardiovascular and cerebrovascular diseases	752	25	Myocardial ischemia/reperfusion injury (MIRI), cerebral ischemia/stroke, atherosclerosis, heart failure, hypertension, thrombosis
Malignant tumors	601	20	Liver cancer, lung cancer, breast cancer, colorectal cancer, gastric cancer, leukemia, glioma
Metabolic diseases	448	15	Type 2 diabetes mellitus, diabetic nephropathy/retinopathy, non-alcoholic fatty liver disease (NAFLD), obesity, hyperlipidemia, gout
Neuropsychiatric disorders	446	15	Alzheimer’s disease (AD), Parkinson’s disease (PD), depression, anxiety, neuropathic pain
Autoimmune/Inflammatory diseases	398	13	Rheumatoid arthritis (RA), ulcerative colitis (UC), systemic lupus erythematosus (SLE), asthma, psoriasis, atopic dermatitis
Infectious diseases	197	7	Influenza, hepatitis B, *Helicobacter pylori*, drug-resistant bacteria, COVID-19
Others (bone, kidney, liver, eye, skin diseases, *etc.*)	170	5	Osteoporosis, liver/kidney fibrosis, diabetic retinopathy, psoriasis, chronic nephritis, overactive bladder

The remaining 1,562 projects without specific disease indications were mainly in fundamental research directions such as CMM, resources; CMM, identification, and CMM, property theory.

#### 3.3.2 Technologies and methods

From 2015 to 2024, the application of technologies and methods in the CMM field exhibited an overall characteristic of “traditional technology as the foundation, rapid penetration of modern technology, and swift emergence of cutting-edge technology,” rapidly iterating and advancing from traditional chemical analysis and pharmacological techniques towards diversified directions such as multi-omics, systems biology, cutting-edge biotechnologies, and artificial intelligence (Table 5).

**TABLE 5:** Analysis of technology/method types involved in completed projects in CMM field of NSFC from 2015 to 2024.

Technology/Method category	Number of projects (n)	Proportion (%)	Representative techniques
Omics technologies	598	20	Metabolomics (LC-MS/GC-MS), proteomics (iTRAQ/TMT), transcriptomics (RNA-seq), lipidomics
Pharmacodynamic substance discovery and screening	447	15	Affinity chromatography, ultrafiltration mass spectrometry, cell membrane chromatography, molecular imprinting, targeted fishing, fluorescent probes
Molecular biology and gene editing	396	13	CRISPR/Cas9, gene knockout/overexpression, RNA interference, qPCR, western blot, immunofluorescence, microdialysis
Network pharmacology and systems biology	352	12	Network construction, molecular docking, virtual screening, pathway enrichment analysis, multi-target prediction
Gut microbiota research	298	10	Fecal microbiota transplantation (FMT), metagenomic sequencing, 16S rRNA, germ-free animals, short-chain fatty acid detection
High-resolution Mass spectrometry and imaging	249	8	UPLC-Q-TOF, MALDI-TOF, mass spectrometry imaging (MSI), ion mobility, LC-MS/MS
Formulation and drug delivery systems	247	8	Nanoparticles, liposomes, microemulsions/gels, exosomes, pickering emulsions, 3D printing
Identification and quality control	198	7	DNA barcoding, near-infrared spectroscopy (NIRS), surface-enhanced Raman spectroscopy (SERS), electrochemical sensors, immunochromatography, electronic nose
Other cutting-edge technologies	195	7	Single-cell sequencing, organoids, microfluidic chips, 3D bioprinting, artificial intelligence/deep learning, spatial transcriptomics

Omics technologies were the most widely applied modern technologies, accounting for 20%, including metabolomics, proteomics, transcriptomics, etc., serving as the core technological foundation for promoting the precision-oriented transformation of CMM research. Pharmacodynamic substance discovery and screening technologies, molecular biology and gene editing technologies, network pharmacology and systems biology technologies, and gut microbiota research technologies were core applied technologies, accounting for 15%, 13%, 12%, and 10%, respectively. They are widely used in active ingredient screening from CMM, mechanism elucidation, and research on the compatibility principles of CMM formulas. Additionally, high-resolution mass spectrometry and imaging, preparation and drug delivery systems, and identification and quality control technologies were core supporting technologies. The application scale of cutting-edge technologies such as single-cell sequencing, organoids, and artificial intelligence is growing rapidly, demonstrating strong development potential (Ni et al., 2025; Chen et al., 2026; Zhang et al., 2025; Xie et al., 2025; Liu X. Y. et al. 2025; Chen W. et al. 2025).

#### 3.3.3 Research mechanisms

From 2015 to 2024, the research mechanisms in the CMM field exhibited an overall characteristic of “deepening from macroscopic efficacy to microscopic mechanisms, expanding from single pathways to multi-dimensional regulation, and extending from classical mechanisms to emerging mechanisms.” Research depth continuously increased, research scope constantly expanded, forming a multi-dimensional, multi-level mechanism research system (Table 6).

**TABLE 6:** Analysis of research mechanism types involved in completed projects in CMM field of NSFC from 2015 to 2024.

Mechanism category	Number of projects (n)	Proportion (%)	Representative pathways/Regulatory
Inflammation and immune regulation	596	20%	NF-κb pathway, NLRP3 inflammasome, MAPK pathway; macrophage M1/M2 polarization; T cell subsets (Th17/Treg, CD8^+^); pro-/anti-inflammatory cytokines
Signal transduction pathways	594	20%	PI3K/AKT/mTOR, wnt/β-catenin, JAK/STAT, TGF-β/Smad, MAPK/ERK, AMPK, hedgehog, notch
Regulation of cell death modes	498	17%	Apoptosis (Bax/Bcl-2/Caspase), autophagy (LC3/Beclin1/mTOR), pyroptosis (NLRP3/GSDMD), ferroptosis (GPX4/ACSL4), necroptosis (RIPK1/3)
Regulation of oxidative stress and mitochondrial function	397	13%	ROS scavenging, Nrf2/ARE pathway, antioxidant enzymes (SOD/CAT); mitochondrial membrane potential, mitochondrial dynamics (fusion/fission), ATP synthesis, respiratory chain complexes
Regulation of gut microbiota and metabolic interaction	299	10%	Gut-brain axis, gut-liver axis, gut-lung axis; short-chain fatty acids, bile acid metabolism, tryptophan metabolism; TMAO, LPS/TLR4 pathway
Epigenetic regulation	248	8%	DNA methylation, histone modification (acetylation/methylation), non-coding RNAs (mirna/lncrna/circrna), m6A modification, chromatin remodeling
Metabolic reprogramming regulation	197	7%	Glycolysis (HK2/PKM2), fatty acid oxidation (PPARα/CPT1), glutaminolysis; observed in tumors, diabetes, cardiomyopathy, immune cell polarization
Organelle and subcellular mechanism regulation	148	4.93%	Endoplasmic reticulum stress (PERK/atf6/ire1), mitophagy (PINK1/Parkin), lysosomal function, cytoskeletal remodeling, lipid rafts

Inflammation and immune regulation, along with signal transduction pathways, were the two most concentrated mechanism directions, each accounting for 20%. The former focuses on the NF-κB pathway, NLRP3 inflammasome, immune cell polarization, *etc.*, representing the fastest-growing mechanism research direction in recent years. The latter covers classical signaling pathways such as PI3K/AKT/mTOR and Wnt/β-catenin, constituting the classic core direction for studying the mechanisms of CMM. Regulation of cell death modes, regulation of oxidative stress and mitochondrial function, and regulation of gut microbiota and metabolic interaction were core mechanism directions, accounting for 17%, 13%, and 10%, respectively. Among these, novel cell death modes like ferroptosis and pyroptosis, as well as gut-target axis interaction regulation, have emerged as recent hotspots (Yang et al., 2026; Wei et al., 2025; Gao et al., 2025; Du et al., 2025). Additionally, epigenetic regulation, metabolic reprogramming regulation, and organelle and subcellular mechanism regulation are emerging expanding directions, with research depth continuously increasing (Xu et al., 2024; Niu and Du, 2026; Liu Y. Z. et al. 2025).

#### 3.3.4 Research models

From 2015 to 2024, the research models in the CMM field spanned the entire chain, from CMM crude materials/processed pieces, CMM formulas/patented medicines, CMM extracts/active fractions, CMM monomers/constituents, to ethnic medicines, novel formulations, and cell/animal models, forming a complete research system (Table 7).

**TABLE 7:** Analysis of research model types involved in completed projects in CMM field of NSFC from 2015 to 2024.

Research Model	Number of Projects (n)	Proportion (%)	Representative Models/Application Directions
CMM monomers/Constituents	897	30	Ginsenosides, tanshinones, berberine, curcumin, artemisinin, baicalin, geniposide, schisandrin B; used for target discovery, in-depth mechanism study, structural modification
CMM formulas/Patented medicines	748	25	Classical formulas (huang-lian-jie-du-tang, liu-wei-di-huang-wan, bu-yang-huan-Wu-tang, Si-Ni-san); modern preparations (danhong injection, shexiang baoxin pill, shufeng jiedu capsule); used for compatibility mechanism research, overall efficacy evaluation
CMM extracts/Active fractions	596	20	Total flavonoids, total saponins, total alkaloids, total phenolic acids, polysaccharides; balancing holism and operability, used for preliminary screening, bioactivity-guided isolation
CMM crude materials/Processed pieces	447	15	Single herbs (*Salvia miltiorrhiza* bunge, *Astragalus membranaceus* (fisch.) bunge, *Panax ginseng* C.A.Mey., *Angelica sinensis* (oliv.) diels, *Rehmannia glutinosa* (gaertn.) steud.); processed products (stir-fried with wine/vinegar/salt/honey); used for resource evaluation, processing mechanism, geoherbalism studies
Ethnic medicines	298	10	Tibetan medicines (rhodiola rosea, terminalia chebula, herpetospermum caudigerum); Mongolian medicines (Sengdeng-4, Shudage-4); miao medicines (periploca forrestii, polygonum capitatum, wikstroemia indica); Uyghur, dai, Zhuang, tujia medicines; used for developing characteristic resources, validating traditional efficacy

Botanical names in this table follow the formatting rule: italic for genus and species, plain text for taxonomic authority, validated via WFO and MPNS.

CMM monomers/constituents were the most widely applied research model, involved in 897 projects (30%), including active constituents such as ginsenosides, tanshinones, and berberine, serving as the core model for basic CMM research and new drug development. CMM formulas/patented medicines were the second largest research model, involved in 748 projects (25%), covering classical formulas and modern patented medicines, acting as the core model for inheriting and innovating TCM theory. CMM extracts/active fractions, crude materials/processed pieces, and ethnic medicines were core basic models, accounting for 20%, 15%, and 10%, respectively. Ethnic medicine represents a uniquely characteristic research direction, with sustained activity in developing characteristic resources and innovative research. Furthermore, the application scale of novel research models such as nano formulations, exosome carriers, and organoid models is growing rapidly, becoming emerging research directions in recent years and promoting the rapid expansion of CMM research towards precision-oriented models (Zhao et al., 2026; Chen et al., 2026).

#### 3.3.5 Research characteristics and trends across different periods

Using the comprehensive reform of the Department of Health Sciences and the official implementation of the new application code system in 2020 as a key dividing point (National Natural Science Foundation of China, 2020; Sun et al., 2022), this study divided the CMM research landscape from 2015 to 2024 into three periods: pre-reform period (2015–2017), reform transition period (2018–2020), and reform deepening period (2021–2024), and conducted a time-series analysis. As shown in Table 8, overall, the CMM research from 2015 to 2024 exhibited a clear three-stage development trajectory, achieving a leap from traditional experience-based basic research to systems biology-driven mechanistic research, and then to precision-oriented, diversified research empowered by cutting-edge technologies. The level of discipline modernization continuously improved, and research depth and breadth continuously expanded.

**TABLE 8:** Analysis of research characteristics and trends of completed projects in CMM field of NSFC from 2015 to 2024.

Dimension	Pre-reform Period (2015–2017)	Reform Transition Period (2018–2020)	Reform Deepening Period (2021–2024)
Overall characteristics	Dominated by traditional resources and basic pharmacology; methods focused on chemical analysis and classical molecular biology	Rise of systems biology and multi-omics; gut microbiota, exosomes, and autophagy became hotspots	Driven by cutting-edge technologies; focus on novel cell death modes, metabolic reprogramming, epigenetics, targeted drug delivery
Disease types	Cardiovascular/cerebrovascular diseases and tumors dominant; metabolic and neuropsychiatric disorders began to grow	Significant increase in metabolic diseases (diabetes, NAFLD) and neuropsychiatric disorders (AD, depression)	Metabolic diseases, neuropsychiatric disorders, and tumors equally emphasized; autoimmune diseases remain active
Technologies and methods	Extraction and isolation, HPLC fingerprinting, simple efficacy models, western blot, PCR	Metabolomics, network pharmacology, gut microbiota sequencing, exosome isolation, emergence of CRISPR	Single-cell sequencing, spatial transcriptomics, organoids, microfluidics, AI/deep learning, nano-drug delivery
Research mechanisms	Apoptosis, oxidative stress, inflammation (NF-κB), simple signaling pathways (PI3K/AKT, MAPK)	Autophagy, ER stress, non-coding RNAs, epigenetics (methylation), immune regulation (macrophage polarization)	Ferroptosis, pyroptosis, metabolic reprogramming, post-translational modifications (lactylation/SUMOylation), gut-X axis, T cell exhaustion
Research models	Primarily monomers/constituents, formulas, crude materials/processed pieces	Increase in extracts/active fractions; active ethnic medicine research	Equal emphasis on monomers/constituents and formulas; emergence of nanoformulations, exosome carriers, organoid models

## 4 Discussion

This systematic analysis of completed NSFC projects in the CMM field from 2015 to 2024 comprehensively reveals the completion status, research outputs, four-echelon classification, and research characteristics of China’s CMM field over the past decade. It provides comprehensive empirical data support and decision-making references for the subsequent optimization of scientific research resource allocation, adjustment and improvement of NSFC funding strategies, and high-quality inheritance, innovation, and development of the discipline.

### 4.1 Comparison with previous related studies

Compared with previous studies focusing on NSFC funding applications or single sub-directions in the CMM field, the innovations and advantages of this study are mainly reflected in three aspects: First, the comprehensiveness of the research object. Most previous studies focused on the application and funding status of NSFC projects. In contrast, this study takes the full set of completed projects in the CMM field from 2015 to 2024 as the research object, representing the largest-scale and most comprehensive systematic analysis of completed projects in the CMM field to date, capable of more truly and comprehensively reflecting the actual outputs and development characteristics of basic research in CMM. Second, the systematic nature of the research dimensions. Previous studies mostly focused on the geographical distribution of projects, supporting institutions, applicant profiles, or development characteristics of single sub-directions. This study, however, conducted systematic multi-dimensional analyses from core perspectives such as research output, translational potential, disease types, technical methods, research mechanisms, research models, and temporal trends, comprehensively revealing the in-depth characteristics and evolutionary patterns of research in the CMM field. The research content is more systematic and comprehensive. Third, the targeted timing of research nodes. Using the 2020 reform of the NSFC discipline code system as a dividing point, a three-stage time-series comparative analysis was conducted, clarifying the impact of disciplinary layout reform on field development and providing more targeted empirical evidence for subsequent adjustments to funding strategies.

### 4.2 Existing problems and challenges in the development of the CMM field

The analytical results of this study indicate that from 2015 to 2024, basic research in China’s CMM field achieved significant development, with sustained growth in project scale, steady improvement in research output, rapid iteration of technical methods, and increasing diversification of research directions. However, several problems and challenges urgently need to be addressed, mainly reflected in the following aspects:

First, there is a systemic shortfall in the modern scientific elucidation of classical TCM theories. Currently, systematic and multi-dimensional research on the scientific connotations of core TCM concepts such as body constitution, pathogenesis, and therapeutic principles is lacking. This shortfall directly leads to the long-term fragmentation of research on the compatibility mechanisms of CMM formulas at the level of “herb pair-pathway” validation, making it difficult to form a complete theoretical system or adequately support the scientific elucidation of CMM formula compatibility, thereby constraining the modern development of TCM theory.

Second, the depth and breadth of innovative application of new technologies and methods are insufficient, and their enabling role is not fully realized. Although new technologies such as artificial intelligence, synthetic biology, and systems biology have achieved preliminary applications in the CMM field, they remain concentrated in a few sub-directions. Their application across the entire chain, including CMM resource protection, quality control, formula compatibility analysis, innovative drug development, and clinical efficacy evaluation, is insufficient to fully enable the modernization of CMM, restricting the precision-oriented and intelligent upgrading of CMM research.

Third, the efficiency of research translation is low, and the actual industrialization rate of patents is insufficient. Over the past decade, 2,811 patents have been generated in the CMM field, with an average potential conversion ratio of 61%. However, most patents remain at the authorization stage without achieving industrialization or clinical translation. The underlying reasons include: basic research primarily focuses on monomers or simple formulas, which is disconnected from actual industrial needs; patent technologies lack support from drug-likeness evaluation and scale-up studies.

Fourth, research homogenization is prominent, and distinctive, original research is insufficient. Significant homogenization is evident in disease research, technical methods, and mechanism elucidation. A large number of projects concentrate on the validation of classical signaling pathways (e.g., the 5 pathways like PI3K/AKT, NF-κB) and the repetitive application of popular techniques (e.g., network pharmacology, 16S rRNA sequencing). Original research based on TCM’s characteristic theories and resources is insufficient. Low-level repetitive research seriously constrains the improvement of the discipline’s core competitiveness. Notably, this phenomenon is associated with the current NSFC project review mechanism: the typical 3–4 years execution period for General Projects and Young Scientist Fund Projects objectively encourages researchers to choose technically mature, lower-risk research paths that facilitate rapid publication, creating an implicit disincentive for exploring original innovations requiring longer cycles and higher risks.

### 4.3 Countermeasures and suggestions for promoting high-quality development of the CMM field

To address the core issues mentioned above and based on the data analysis results, the following core countermeasures are proposed to promote high-quality disciplinary development:

First, strengthen the systematic modern elucidation of classical TCM theories and construct a multi-dimensional theoretical analysis system. Focusing on core theories such as TCM body constitution, pathogenesis, and therapeutic principles, it is recommended that the NSFC establish a major research plan titled “Modernization of TCM Theory.” This plan should integrate cutting-edge technologies such as systems biology, multi-omics, and artificial intelligence to conduct multi-dimensional, systematic scientific elucidation, clarify the biological basis of classical theories, provide theoretical support for CMM formula compatibility analysis and innovative drug development, and promote the modern development of TCM theory. The proposed funding intensity should be no less than 5 million RMB per project, with an execution period of 5–8 years.

Second, promote the full-chain innovative application of new technologies and methods to empower the entire process of CMM modernization. Increase funding support for the application of new technologies such as artificial intelligence, synthetic biology, and systems biology in the CMM field. Promote their in-depth application across the entire chain, including CMM resource protection, quality control, formula compatibility analysis, active ingredient screening and synthesis, innovative drug development, and clinical efficacy evaluation. Construct a new technology-empowered CMM modern research system to enhance the precision and intelligence of CMM research.

Third, improve the full-chain support system for innovative drug development in CMM to comprehensively enhance translational efficiency. Establish a special fund for innovative CMM drug development. Construct a full-chain support system covering basic research, active ingredient screening, drug-likeness evaluation, preclinical research, and clinical trials. Focus on supporting innovative CMM formulas, innovative active ingredient drugs, and novel formulation development, addressing core challenges such as the difficulty in elucidating the compatibility mechanisms of formulas and poor drug-likeness of active ingredients. Establish a funding mechanism that bridges basic research and translational application, providing subsequent rolling support for completed projects with good translational prospects. Additionally, reduce the weight of publication count and increase the weight of outcomes such as patent translation and clinical application in project reviews.

Fourth, optimize differentiated tiered funding portfolios adapted to the research characteristics of various CMM subfields and diversified commercial transformation objectives to alleviate homogenized low-level research. Formulate targeted funding strategies matching the four echelons classified by output and conversion performance. For High-Output High-Conversion subfields with mature industrialization prospects, prioritize funding for cross-industry research oriented to new drug R&D commercial goals; for Low-Output Low-Conversion pharmacological subfields with high innovation risks, set up youth original innovation special funds to encourage exploratory research with long cycles; for characteristic ethnic pharmacology and CMM quality evaluation with unique resource advantages, design exclusive funding schemes aligned with their distinctive research and translational demands, so as to realize balanced, high-quality development of all sub-disciplines.

### 4.4 Research limitations

This study relies solely on the NSFC database and does not include data from other funding systems (e.g., Ministry of Science and Technology, National Administration of Traditional Chinese Medicine), making it difficult to fully and promptly reflect the entire research ecosystem. The current discussion only compares the work within China NSFC-related CMM literature, lacking cross-regional comparison of studies from Europe, U.S. or Japan, etc. The study’s definition of “research output” is limited to five categories (journal articles, conference papers, patents, awards, monographs) and does not include terminal effect indicators of clinical translation (e.g., actual application rate of technology in medical institutions, data on improved clinical diagnosis and treatment efficiency). Using only “patent count” to calculate the “potential conversion ratio” cannot fully measure the actual application value of outputs, as some patents may remain only at the authorization stage without achieving industrialization or clinical translation. Hence, Patent count cannot reflect actual clinical or industrial transformation success and acknowledge that this indicator maybe overestimates real translational capacity. Furthermore, cross-disciplinary projects related to CMM within code H3402 (Natural Product Chemistry) were not included, potentially underestimating the overall funding scale and output.

## 5 Conclusion

This study comprehensively reveals the development dynamics, research output characteristics, and disciplinary evolution patterns of basic research in China’s CMM field over the past decade. It provides comprehensive empirical data support and decision-making references for the subsequent optimization of scientific research resource allocation, adjustment and improvement of NSFC funding strategies, and formulation of disciplinary development strategies. It can also serve as a reference for researchers in the CMM field for research topic selection, grasping research frontiers, and clarifying research directions.

## Data Availability

The original contributions presented in the study are included in the article/supplementary material, further inquiries can be directed to the corresponding authors.

## Glossary

AD: Alzheimer’s disease
AKT: Protein kinase B
AMPK: AMP-activated protein kinase
CAGR: Compound annual growth rate
CMM: Chinese Materia Medica
CRISPR: Clustered regularly interspaced short palindromic repeats
FMT: Fecal microbiota transplantation
GC-MS: Gas chromatography-mass spectrometry
GPX4: Glutathione peroxidase 4
HF: Heart failure
HK2: Hexokinase 2
iTRAQ: Isobaric tags for relative and absolute quantification
JAK: Janus kinase
LC-MS: Liquid chromatography-mass spectrometry
LPS: Lipopolysaccharide
MIRI: Myocardial ischemia/reperfusion injury
m6A: N6-methyladenosine
mTOR: Mammalian target of rapamycin
NAFLD: Non-alcoholic fatty liver disease
NF-κB: Nuclear factor-κB
Nrf2: Nuclear factor erythroid 2-related factor 2
NSFC: National Natural Science Foundation of China
PD: Parkinson’s disease
PI3K: Phosphatidylinositol 3-kinase
PKM2: Pyruvate kinase M2
qPCR: Quantitative real-time polymerase chain reaction
RA: Rheumatoid arthritis
RNA-seq: RNA sequencing
SLE: Systemic lupus erythematosus
TCM: Traditional Chinese Medicine
TGF-β: Transforming growth factor-β
TMAO: Trimethylamine N-oxide
TMT: Tandem mass tag
UC: Ulcerative colitis
UPLC-Q-TOF: Ultra-performance liquid chromatography-quadrupole time-of-flight mass spectrometry
WFO: World Flora Online
MPN: Medicinal Plant Names Services

## References

[B1] Cai W. Gong Z. P. Chai Z. (2025). Research progress of supramolecular chemistry in Chinese materia medica based on NSFC-Funded projects. Chin. Tradit. Herb. Drugs 56 (3), 753–759.

[B2] Chen J. Lin C. Z. Fu J. J. (2014). Review on application and funding of NSFC projects in active substances of Chinese materia medica in 2013. World Sci. technol.-mod. Tradit. Chin. Med. 16 (2), 211–215.

[B3] Chen Y. J. Bassi G. S. Wang Y. Yang Y. Q. (2022). Research hotspot and frontier analysis of traditional Chinese medicine in asthma using bibliometric methods from 1991 to 2021. J. Allergy Clin. Immunol. Glob. 1 (4), 185–197. 10.1016/j.jacig.2022.07.004 37779535 PMC10509992

[B4] Chen W. Zhang W. D. Zhao Q. (2025). Application and progress of organ-on-a-chip technology in Chinese materia medica research. Chin. J. Inf. Traditional Chin. Med. 32 (11), 187–192. 10.19879/j.cnki.1005-5304.202502116

[B5] Chen Y. H. Zhang D. Xia Y. W. (2025). Visualization analysis of current status, hotspots and trends of traditional Chinese medicine in carotid atherosclerosis based on CiteSpace and VOSviewer. World Sci. technol.-mod. Tradit. Chin. Med. 27 (2), 498–507.

[B6] Chen J. F. Wang M. M. Wu C. (2026a). Research progress of self-assembled peptides delivering active ingredients of Chinese materia medica for anti-tumor, antibacterial and wound healing. Mod. Chin. Med. 28 (2), 378–386. 10.13313/j.issn.1673-4890.20240731003

[B7] Chen Z. Chen Z. Lu Z. Q. (2026b). Research progress of nano-delivery system of active ingredients from Chinese materia medica in the treatment of kidney diseases. China J. Chin. Mater. Med. 1–16. 10.19540/j.cnki.cjcmm.20260430.303 42693040

[B8] Dai Z. Liao X. (2021). Hospital-based health technology assessment: the next frontier for traditional Chinese medicine hospitals. J. Tradit. Chin. Med. Sci. 8 (2), 110–114. 10.1016/j.jtcms.2021.03.002

[B9] Du Y. S. Luo L. Han B. W. (2025). Research progress of traditional Chinese medicine regulating intestinal flora in the treatment of non-alcoholic fatty liver disease based on “gut-microbiota-liver axis”. Shaanxi J. Traditional Chin. Med. 46 (6), 857–860.

[B10] Gao W. Guo S. Z. Han L. W. Zhang F. Z. (2016). Analysis of funded and completed projects in Chinese materia medica resources supported by NSFC in recent three years. China J. Chin. Mater. Med. 41 (19), 3696–3701. 10.4268/cjcmm20161932 28925170

[B11] Gao X. Liu C. Tong D. P. (2025). Research progress of traditional Chinese medicine in the treatment of Parkinson’s disease based on “microbiota-gut-brain axis” theory. Chin. J. Exp. Tradit. Med. Formulae. 1–13. 10.13422/j.cnki.syfjx.20260236

[B12] Han L. Lin C. Z. Zhao H. X. (2022). Analysis of funded projects in toxicology of Chinese materia medica supported by NSFC from 2012 to 2021. China J. Chin. Mater. Med. 47 (5), 1415–1420. 10.19540/j.cnki.cjcmm.20211228.401 35343171

[B13] Liu X. Y. Lu Y. C. Han L. (2025). Research progress of tumor organoids in anti-tumor therapy of monomer components of Chinese materia medica. Anhui Med. Pharm. J. 29 (9), 1729–1733.

[B14] Liu Y. Z. Song F. L. Wang N. (2025). Mechanism of traditional Chinese medicine in prevention and treatment of MAFLD from the perspective of organelle crosstalk. Mod. Dig. and Intervention 30 (10), 1093–1096.

[B15] Ma T. C. Cao M. K. Wang G. F. (2011). Basic research competitiveness of provinces and cities in China: evidence from the national natural science foundation of China. Sci. Bull. 56 (36), 3115–3121. 10.1360/972011-1819

[B16] National Natural Science Foundation of China (2020). National Natural Science Foundation of China Application Codes. 2020 Edition. China, Beijing: National Natural Science Foundation of.

[B17] National Natural Science Foundation of China (2025). NSFC big data knowledge management service platform: data statistics [EB/OL]. Available online at: https://kd.nsfc.cn/supportStatistic (Accessed May 26, 2026).

[B18] Ni P. Yang Y. W. Tang H. K. (2025). Research progress of AUF-LC/MS in Chinese materia medica analysis in recent five years. Mod. Chin. Med. 27 (9), 1788–1796. 10.13313/j.issn.1673-4890.20240927001

[B19] Niu Y. Y. Du M. R. (2026). Research progress of active ingredients of Chinese materia medica regulating macrophage glycolysis reprogramming in metabolic diseases. Lishizhen Med. Mater. Med. Res. 1–11. Available online at: https://link.cnki.net/urlid/42.1436.R.20260522.1704.008 (Accessed May 31, 2026).

[B20] Peng Y. Wang M. Chen T. Wang L. A. Huang T. (2012). Statistical analysis of NSFC-Funded projects in Chinese materia medica from 2002 to 2011. Bull. Natl. Nat. Sci. Found. China 26 (5), 307–310.

[B21] Shang H. C. Huang J. L. Han L. W. Pei L. P. Guo L. Lin N. (2011). Analysis on application and funding of traditional Chinese medicine projects supported by NSFC in 2010. J. Chin. Integr. Med 9 (10), 1045–1050. 10.3736/jcim20111001 22015182

[B22] Shen J. K. Zhang Y. Q. Li H. N. (2022). Research hotspots of Chinese materia medica and natural medicines in China based on scientometrics and visualization. Chin. Pharm. Aff. 36 (1), 92–104. 10.16153/j.1002-7777.2022.01.012

[B23] Sun L. Wang J. J. Fan Y. J. (2022). Practice and thinking of NSFC reform on adjusting application codes to optimize disciplinary layout in the new era. Bull. Natl. Nat. Sci. Found. China 36 (5), 693–700. 10.16262/j.cnki.1000-8217.2022.05.005

[B24] Tan X. N. Wu W. R. Lai H. L. (2021). Funding status and research hotspots of Chinese materia medica identification supported by NSFC from 2010 to 2019. Chin. J. Inf. Traditional Chin. Med. 28 (8), 22–28. 10.19879/j.cnki.1005-5304.202006096

[B25] Wang J. W. Liu Y. Tong Y. Y. Yang C. Li H. Y. (2016). Hotspots and trends of molecular pharmacognosy projects in Chinese materia medica (H28) funded by NSFC. China J. Chin. Mater. Med. 41 (9), 1617–1621. 10.4268/cjcmm20160908 28891608

[B26] Wei Y. X. Han J. P. Yang Y. F. (2025). Research progress of traditional Chinese medicine regulating ferroptosis in hematological malignancies. Chin. J. Pathophysiol. 41 (4), 809–815.

[B27] Wen Y. L. Zhang W. W. Qin L. L. Huang Y. L. Chen Y. F. Wang X. (2023). Analysis of funding and research hotspots of traditional Chinese medicine projects supported by the national natural science foundation of China from 2011 to 2020. Eval. Analysis Drug Use Chin. Hosp. 23 (9), 1041–1045. 10.14009/j.issn.1672-2124.2023.09.002

[B28] Wu L. F. Liu J. Q. Liu Z. Y. (2025). Bibliometric analysis of research hotspots and trends in Chinese materia medica analysis. Hebei J. Ind. Sci. Technol. 42 (4), 365–382.

[B29] Xie W. X. Zhang Z. X. Liu Y. (2025). Application and research progress of artificial intelligence in quality control of Chinese materia medica. Chin. Tradit. Herb. Drugs 56 (15), 5616–5631.

[B30] Xu X. Y. Zhu Y. P. Liu Y. Q. (2024). Research progress on mechanism of traditional Chinese medicine against hepatocellular carcinoma based on epigenetic regulation. Chin. J. Exp. Tradit. Med. Formulae 30 (23), 281–291. 10.13422/j.cnki.syfjx.20250391

[B31] Yang P. L. Zhao Y. Q. Lu M. J. (2026). Research progress of traditional Chinese medicine targeting intracellular redox balance to improve ferroptosis in hypertension. World Chin. Med. 21 (1), 162–170.

[B32] Zhang M. X. Guo R. (2013). From transformation rate of scientific and technological achievements to transformation efficiency: indicator system design and empirical analysis. Soft Sci. 27 (12), 85–89. 10.3969/j.issn.1001-8409.2013.12.019

[B33] Zhang Y. Y. Zeng Y. Z. Zhong H. S. (2025). Research progress of biomimetic nano-drug delivery system of active ingredients from Chinese materia medica in liver diseases. Chin. J. New Drugs 34 (10), 1082–1089. 10.20251/j.cnki.1003-3734.2025.10.012

[B34] Zhao J. X. Zhang D. M. Li C. Geng Z. H. Chen C. He Y. H. (2025). Analysis on application and funding of traditional Chinese medicine research projects for chronic skin ulcers supported by NSFC during 2008–2022. Chin. J. Basic Med. Traditional Chin. Med. 31 (6), 1120–1124.

[B35] Zhao Z. R. Long L. Z. Fu C. G. (2026). Research progress of modern nano-formulations of Chinese materia medica in cardiovascular diseases. Chin. J. Integr. Med. Cardio-Cerebrovasc. Dis. 24 (10), 1537–1541.

